# Evolution of interventional endoscopic ultrasound

**DOI:** 10.1093/gastro/goad038

**Published:** 2023-06-30

**Authors:** Mark J Radlinski, Daniel S Strand, Vanessa M Shami

**Affiliations:** Division of Gastroenterology and Hepatology, University of Virginia, Charlottesville, VA, USA; Division of Gastroenterology and Hepatology, University of Virginia, Charlottesville, VA, USA; Division of Gastroenterology and Hepatology, University of Virginia, Charlottesville, VA, USA

**Keywords:** endoscopic ultrasound, interventional endoscopic ultrasound, biliary drainage, gastrojejunostomy, pancreatic pseudocyst drainage

## Abstract

Endoscopic ultrasound (EUS) has become an indispensable modality for the assessment of the gastrointestinal tract and adjacent structures since its origin in the 1980s. Following the development of the linear echoendoscope, EUS has evolved from a purely diagnostic modality to a sophisticated tool for intervention, with numerous luminal, pancreaticobiliary, and hepatic applications. Broadly, these applications may be subdivided into three categories: transluminal drainage or access procedures, injection therapy, and EUS-guided liver interventions. Transluminal drainage or access procedures include management of pancreatic fluid collection, EUS-guided biliary drainage, EUS-guided bile duct drainage, EUS-guided pancreatic duct drainage, and enteral anastomosis formation. Injection therapies include therapeutic EUS-guided injections for management of malignancies accessible by EUS. EUS-guided liver applications include EUS-guided liver biopsy, EUS-guided portal pressure gradient measurement, and EUS-guided vascular therapies. In this review, we discuss the origins of each of these EUS applications, evolution of techniques leading to the current status, and future directions of EUS-guided interventional therapy.

## Introduction

Endoscopic ultrasound (EUS) has become an indispensable modality for the assessment of the gastrointestinal tract and adjacent structures since its origin in the 1980s [1, 2]. Although initially a purely diagnostic instrument, EUS has evolved into a sophisticated and versatile tool for intervention with numerous luminal and pancreatobiliary applications. Broadly, interventional EUS may be subdivided into three categories: transluminal drainage or access procedures, injection therapy, and EUS-guided liver applications. In this review, we will discuss the evolution, current status, and future directions of EUS-guided interventional therapy.

## Pancreatic fluid collection

The local complications of pancreatitis, including pancreatic fluid collections (PFCs), are a frequent clinical problem that engages the attention of the practicing gastroenterologist [3]. Rogers *et al.* [4] published the first report of an endoscopically drained pancreatic fluid collection at the University of Chicago in 1973. Using a forward-viewing endoscope, a large 10-cm hemispherical gastric bulge was identified. A 21-gauge needle attached to Teflon tubing was used to puncture and aspirate 45 ml of cloudy brown fluid. The patient did not experience relief and imaging 2 days after the procedure showed the cyst had refilled entirely and “resumed its hemispherical form” [4].

In 1985, Kozarek *et al.* [5] reported transenteric pseudocyst drainage in four patients, all of whom were high-risk surgical patients. They all underwent drainage via fistulotomy using a diathermic needle knife through a duodenoscope. During the procedure, an incision of 0.5–1.5 cm was created. Two patients had pseudocyst resolution (although they required multiple endoscopic fistulotomies). One of these two patients required a nasocystic tube for additional drainage and two were considered endoscopic failures. Of note, one patient had an episode of major bleeding that did not necessitate intervention [5]. In 1987, a case series was published reporting nasocystic catheter insertion in PFCs (following fistulotomy) and saline irrigation [6]. In a subset of 20 patients, there were two perforations and two bleeding events, one of which was fatal. Additionally, patient tolerance of the nasocystic tube was poor secondary to nasal discomfort. Plastic stents were then introduced to maintain drainage. Two case series of 24 and 37 patients reported a 90% cyst resolution with transenteric placement of 7F and 10F stents. Adverse events included five bleeding events, two perforations, and one incidental gallbladder puncture [7]. Overall, “non-EUS” or conventional transmural drainage was limited in the setting of a non-visible bulge (imperfect targeting) and propensity for vascular injury (bleeding events) and perforations [8, 9].

The advent of linear echoendoscopes in the early 1990s revolutionized the process and effectiveness of PFC drainage. In 1992, the two-scope technique was described in which a linear echoendoscope was used to puncture the pancreatic cyst and insert a guide wire. The scope was then exchanged for a duodenoscope to allow a larger working channel (4.2 vs 2.0 mm) and a 10F plastic stent placement. Later, echoendoscopes with 3.8-mm instrument channels became available, simplifying the technique and allowing single-instrument EUS-guided insertion of 10F plastic stents [10, 11]. Further specialized equipment, including introducer systems for cystenterostomy, made EUS drainage more accessible.

Although pseudocysts were the initial target of EUS-directed drainage, by the mid-1990s, other PFCs, such as walled-off necrosis (WON), became of interest to interventional endoscopists due to the morbid nature of traditional surgical treatment [12]. In 1996, the first case of endoscopic pancreatic necrosis therapy by continuous irrigation was described by Baron *et al.* [13]. This case series, in which most patients underwent intra-pancreatic nasobiliary lavage in addition to 10F transmural stent placement, demonstrated that endoscopic necrosectomy was feasible and showed a >50% reduction in pancreatic necrosis on follow-up cross-sectional imaging. Direct debridement of necrotic PFCs first took place in 2000. Three patients who had previously failed EUS-guided transmural stent placement underwent direct transmural access and dilation followed by debridement. Snares and baskets were used in this initial case series for debridement [14].

**Figure 1.**
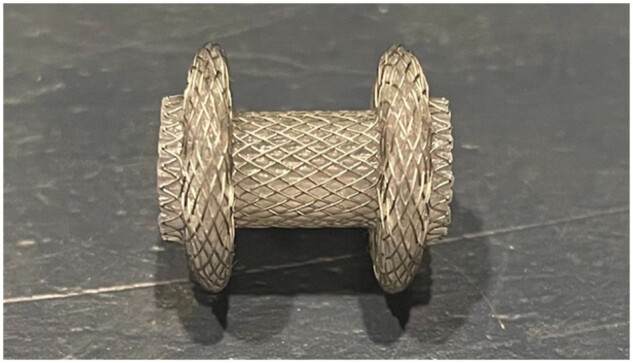
Lumen-apposing metal stents

Concurrently, the commercial development of self-expandable metal stents (SEMS) provided a novel mechanism to create large-diameter transmural PFC entry without the need to stage initial access and dilation. The first (uncovered) SEMS was placed in 1994 in a patient who had failed nasocystic drainage. By 2008, EUS-guided placement of covered SEMS for cystgastrostomy/cystduodenostomy had gained significant traction [15]. A case series of 18 patients demonstrated 95% successful stent placement. Resolution of the targeted fluid collection occurred in 75% of the cases. In addition to the benefit of a larger lumen for drainage and tamponade effect to decrease bleeding, the introduction of the SEMS allowed easier access to the cyst with a gastroscope and endoscopic necrosectomy. However, the use of SEMS was hampered by migration of the transmural stent in up to 40% of patients included in initial reports. Given the high rate of stent migration, lumen-apposing metal stents (LAMS) were developed (in 2004) for transluminal drainage and were first implemented in 2011 (Figure 1). These short, dumbbell-shaped stents with bilateral circumferential flanges allow apposition and fixation of two lumens (or a lumen and adjacent cavity) with a significantly decreased risk of migration [16]. In a review of the safety and adverse events of LAMS by Yang *et al.* [17], there was a reported 4.1% adverse event rate that included self-limited bleeding, stent migration (salvageable during the procedure), and infection (no reported procedure-related mortality or perforation).

**Figure 2.**
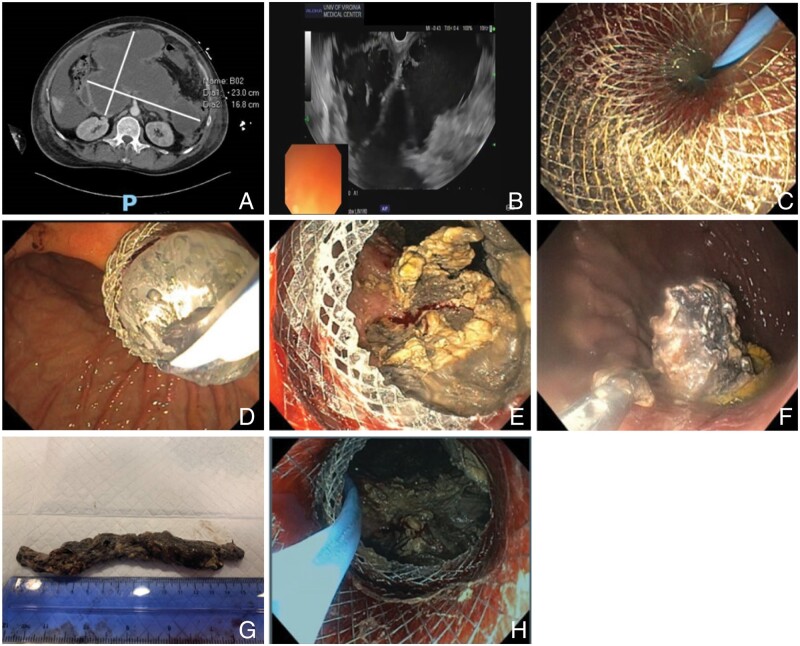
Endoscopic ultrasound (EUS)-directed drainage of pancreatic fluid collections. (A) Axial computed tomography of a pancreatic fluid collection. (B) EUS allows direct visualization of the pancreatic fluid collection. A 19-gauge needle is used to access the cyst and a guide wire is placed in the cavity. (C) The distal end of the lumen-apposing metal stents (LAMS) is placed in the fluid collection using an electrocautery-enhanced delivery system. Next, the proximal/enteral end of the stent is deployed within the echoendoscope and the scope is gradually withdrawn to allow deployment within the stomach or duodenum. (D) Depending on the location of the LAMS and the need for necrosectomy, the size can range from 10 to 20 mm. The tract can then be dilated over the wire with a through-the-scope balloon to allow increased drainage, direct access, and necrosectomy if needed. (E) Typically, the echoendoscope is removed, a gastroscope is introduced, and the cavity can be directly accessed. (F) Various instruments can be used, including snares, baskets, and nets, to debride the cavity. (G) Necrotic pancreas can be systematically removed from the walled-off collection. (H) Double-pigtail plastic stents are placed within the lumen of the LAMS to facilitate drainage and theoretically decrease risk of bleeding and perforation as the cavity contracts.

EUS allows direct drainage of PFCs, often found in the peripancreatic retroperitoneal space next to the stomach and the duodenum (Figure 2). Pseudocysts and simple fluid collections can be drained via direct aspiration and/or, often, plastic double-pigtail stents are placed. LAMS can also be considered for the drainage of complex fluid collections, as this allows the debridement of solid components (e.g. WON) and may require fewer procedures.

Plastic stents are frequently inserted within the LAMS at the endoscopist’s discretion. This practice has enhanced patency and facilitated drainage while decreasing adverse events [18]. Placement of double-pigtail stents also buffers the WON cavity wall away from the LAMS, reducing the risk of mechanically derived adverse events such as delayed bleeding [18]. Typical practice has been to remove the LAMS at 4 weeks following initial drainage and emerging data support the subsequent insertion of double-pigtail plastic at that time, especially in cases of known or suspected complete pancreatic duct disruption [19, 20]. The advent of LAMS has also allowed earlier intervention in PFCs [21, 22]. While there has been good reason for the enthusiasm regarding the use of LAMS for drainage and pancreatic necrosectomy, the universal application of this tool may not translate into hard outcome benefits [23, 24]. In a recent randomized–controlled trial of 60 patients randomized to LAMS versus plastic stents, there was no difference in the number of procedures performed to achieve treatment success (primary outcome defined as resolution of WON on cross-sectional imaging and symptoms resolution at 6 months), length of stay, adverse events, or readmissions [25]. Another single-center randomized–controlled trial of 22 patients with WON showed non-superiority of LAMS versus plastic stents in achieving imaging resolution of necrosis (no difference in technical success, adverse events, length of stay, and mortality as well) [26].

## Drainage, enteral access, and anastomosis formation

Endoscopic retrograde cholangiopancreatography (ERCP) is the gold-standard procedure for biliary drainage. In cases where ERCP cannot be completed, EUS-guided biliary drainage (EUS-BD) can provide an alternative means of biliary decompression. ERCP is not always successful for transpapillary drainage, with reports suggesting a failure of transpapillary biliary access in up to 16% of cases (5%–7% in the US) [27]. Clinical scenarios that may interfere with a successful ERCP include proximal duodenal obstruction (precluding the passage of a duodenoscope), inability to cannulate secondary to obstructing stone or mass, and altered anatomy (foregut surgery, duodenal diverticulum).

The traditional approach to unsuccessful ERCP for biliary drainage typically involves the placement of percutaneous transhepatic biliary drainage (PTBD). Placement of a PTBD catheter confers significant risk, with reported complication rates as high as 40% (catheter dislodgement, occlusion, skin infection, cholangitis, bleeding) [28]. Additionally, this approach is frequently associated with a need for repetitive interventions, which can negatively impact a patient’s quality of life. Surgery, another alternative to ERCP, carries significant potential morbidity and may not be an option for many patients.

Endosonography offers the potential for intervention due to the anatomical relationship of the liver and common bile duct (CBD) with the adjacent upper alimentary tract. Specifically, the biliary tree can be identified and targeted through the stomach or duodenum. Linear EUS can facilitate the creation of an anastomosis between the duodenum and the CBD (choledochoduodenostomy [EUS-CD]) or the stomach and left-sided intrahepatic bile duct (hepaticogastrostomy [EUS-HG]). For EUS-CD, the anastomotic connection typically is created between the distal CBD and the duodenal bulb.

Lopes *et al.* [16] first described EUS-BD in 2003. In this case, the CBD was accessed using a 5 French needle knife under EUS guidance with the passage of a 0.35-inch guide wire. In addition, a 10F plastic stent was placed for CBD drainage. Early EUS-BD procedures utilized plastic stents for drainage, but bile leakage along the stents was common (bile leak/peritonitis occurring in ∼10% of patients with plastic stents) [29]. The introduction of SEMS mitigated the risk of bile leakage, but stent migration (reported as high as 57% in some studies) remained a drawback [30]. A meta-analysis demonstrated high technical success (91.2%–94.1%) of EUS-BD procedures at the cost of a relatively high rate of adverse events (14.5% for EUS-CD and 20.9% for EUS-HG) [31]. The most common events reported were bile leaks (8.5%), cholangitis (4.2%), bleeding (4.1%), and luminal perforation (3.0%). These data revealed a trend toward an improved safety profile using LAMS compared with using other stents (adverse events 10.1% vs 15.9%, *P *=* *0.22), though the use of LAMS was limited at the time of publication. Bi-flanged LAMS allows a more straightforward delivery system for biliary drainage with a relatively lower rate of adverse events [32].

EUS-BD can also provide biliary access without direct drainage via a so-called “rendezvous approach.” Here, endosonography is used to introduce an anterograde guide wire into the biliary tree, which is then captured below the papilla to perform traditional ERCP [28, 32–36]. In this procedure, EUS needle puncture of a dilated bile duct is directed toward the major papilla (right torque in D1) instead of the liver (left torque in D1). When direct drainage is desired (as opposed to rendezvous), puncture, bile aspiration, and cholangiography are followed by advancement of the guide wire toward the liver under fluoroscopic guidance. The choledochoduodenostomy is then formalized by the placement of the desired stent. Typical prostheses employed include electrocautery-enhanced LAMS (6- or 8-mm) or fully covered metal biliary stents (which may require track dilation).

## EUS-guided gallbladder drainage

Patients who present with cholecystitis but are not suitable candidates for laparoscopic cholecystectomy (the gold standard for therapy) pose a significant clinical problem. Historically, these patients have been managed with either cholecystostomy tube placement or ERCP-directed transpapillary stenting of the gallbladder. EUS-guided gallbladder drainage (EUS-GBD) offers an alternative endoscopic technique that is particularly valuable in patients whose gallbladder cannot be cannulated via ERCP. The use of LAMS for EUS-GBD was first introduced in 2015 by Irani *et al.* [37]. The procedure of EUS-GBD involves access of the gallbladder in a transduodenal (possible advantage given less susceptibility to peristalsis, less exposure to solid food with risk of occlusion, and possibly easier to repair than a gastric defect if needed) or transgastric (antral) fashion. Direct drainage is provided using a LAMS or SEMS [37]. In some instances, stone clearance can be achieved following stent placement and additional double-pigtail plastic stents are introduced to facilitate continuous drainage. Technical success for the placement of LAMS for gallbladder drainage appears to be equivalent to a percutaneous cholecystostomy tube (97.4% vs 100%) with a reduction in the number of adverse events (25% vs 77.5%) and decreased need for reintervention (2.6% vs 47.5%) over 1 year [38]. Note that the aggregation of adverse events in this study includes the requirement for drain maintenance over that entire year-long interval. Specific procedure-related adverse events reported include stent occlusion, luminal perforation, and percutaneous tube dislodgement. A prospective, multicenter trial of patients with cholecystitis and EUS-GBD demonstrated that LAMS occlusion occurs in ∼7% of patients, which can require reintervention [39]. Retrospective series have also commented on infrequent but serious side effects, including duodenal perforation (1.6%) and pneumoperitoneum (3.2%) [40]. While not suitable for every patient, EUS-directed gallbladder drainage does provide another effective modality in the armamentarium to treat cholecystitis in patients who are unfit for surgery, those who cannot expect relief via ERCP, and patients who are opposed to external drainage. LAMS are typically left in place for 3–6 weeks and then replaced with plastic double-pigtail stents [38].

## EUS-guided pancreatic duct drainage

In patients with a disconnected pancreatic duct and/or for patients in whom traditional retrograde pancreatic access is not possible, EUS-guided anterograde pancreatic duct access can be considered. This procedure involves targeting a dilated upstream pancreatic duct via EUS with subsequent needle puncture and wire access. EUS-guided pancreaticogastrostomy (EUS-PG) via stent placement can be pursued subsequently, particularly if the downstream duct is obstructed and cannot be traversed. This technique was first described by François *et al.* [41] in a case series of four patients with dilated pancreatic ducts. In 2007, Shami *et al.* [42] described 13 cases involving patients with chronic pancreatitis in whom ERCP had failed to provide proximal pancreatic access. These patients were then offered EUS-PG, which was technically successful in 77% (10/13). There was a subsequent study by Falque *et al.* [43] that had a 92.5% technical success rate of pancreaticogastrostomy in chronic pancreatitis.

In the anterograde approach for EUS-guided pancreatic drainage, direct access to the stomach (or, less likely, the small bowel in altered anatomy) allows enteral drainage of the pancreatic duct. This facilitates the creation of a fistulous tract between the pancreatic duct and the alimentary lumen for decompression. Traditionally, plastic stents are used to stabilize this track, though the diameter and configuration may vary. Additionally, EUS can permit retrograde pancreatic intervention by transmural advancement of a wire across the papilla. Technical success depends on pre-existing anatomy and whether a rendezvous technique is attempted. With an anterograde approach, the technical success rate is ∼89%. Adverse events occur in ∼12% of patients, including post-procedure pain (4%), the onset of acute pancreatitis (2%), perforation (1%), bleeding (3%), or development of a peripancreatic fluid collection (2%) [44]. With EUS-guided pancreatic duct access, there was not a significant difference in adverse events when comparing native pancreatic anatomy to surgically altered anatomy (15% vs 11%) [45]. Contraindications to EUS-guided pancreatic duct drainage included a non-dilated duct (increasing the difficulty and possible adverse event risk), inability to locate the pancreatic duct with EUS, or presence of vasculature that interfered with EUS access [44, 46–48].

## Enteral anastomosis

Enteric anastomosis has historically been considered a surgical procedure. Indeed, prior to the recent development of LAMS, non-surgical opportunities to attach one loop of the bowel to another was performed by a few daring and innovative endoscopists [49]. Now, with purpose-built devices, EUS is frequently employed to this end. The initial series of case reports describing EUS-guided gastrojejunostomy (EUS-GJ) [50–53] were published, including the first multicenter US series of 10 patients by Khashab *et al.* [54] in 2015.

The most common indication for EUS-GJ is malignant gastric outlet obstruction when a duodenal stent is not feasible or appropriate. EUS-GJ can also be considered in benign gastric outlet obstruction and for treating afferent loop syndrome. Described techniques for EUS-GJ are variable but typically involve the initial placement of a guide wire past the obstruction under fluoroscopic guidance. Then, a catheter (or a nasobiliary drain) is advanced over the wire and used to inject contrast and/or dye to confirm placement in the desired jejunal limb. Next, cautery-assisted (cutting current), EUS-guided LAMS is deployed across the gastric–jejunal fistula. This can be performed using a “freehand” technique or with the assistance of a guide wire [55, 56]. The freehand technique can avoid an unfavorable axis of insertion due to the guide wire but does so at the cost of additional safety should there be an issue during LAMS deployment. As a competing intervention alongside surgery and enteral stent placement, outcome data for EUS-GJ have been mixed [57]. As a result, enteral stenting is generally suggested for patients with shorter life expectancies (<3 months) or those in whom transluminal puncture is problematic (i.e. ascites). Surgical gastrojejunostomy is more invasive and has a longer recovery time but has demonstrated better long-term outcomes [58–61]. EUS-GJ is associated with a shorter time to the resumption of oral intake than surgery and appears generally comparable. EUS-GJ also has a shorter recovery time, which is important when considering initiation or resumption of chemotherapy for patients with cancer (the concept of transiently withholding treatment in this scenario to “allow healing” is not clearly defined). While promising, there is a need for a high-quality comparative trial to evaluate the ideal role of EUS-GJ, surgical anastomosis, and enteral stenting [55, 56, 62, 63].

The technical success rate (91%–94%) and clinical success rate (88%–90%) of EUS-GJ are both high, with minimal adverse events (≤12% of patients, including perforation, need for surgery, peritonitis, leakage at anastomosis) [55, 56]. Contraindications for a EUS-GJ approach include the presence of ascites (specifically refractory or malignant ascites), as the presence of ascites predisposes to risk of infection.

**Figure 3.**
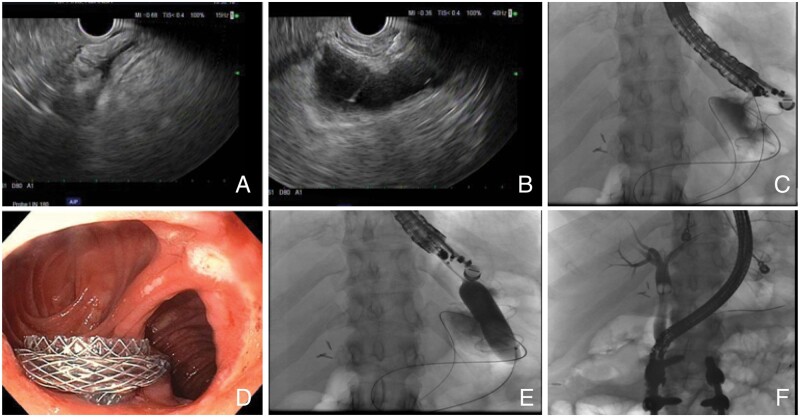
Endoscopic ultrasound (EUS)-directed transgastric endoscopic retrograde cholangiopancreatography (ERCP). (A) In this procedure, the excluded stomach is identified by EUS. The excluded stomach is often described as having a “starfish” appearance. (B) The excluded stomach is accessed using a 19-gauge needle and contrast is injected to confirm location (stomach rugae are visualized fluoroscopically). (C) A stiff wire curled within the excluded stomach. (D) A cautery-assisted catheter is used to deploy a lumen-apposing metal stent, creating a fistulous gastric–gastric tract. (E) The lumen-apposing metal stent (LAMS) is typically dilated between 15 and 20 mm to allow the passage of the duodenoscope. (F) The papilla can then be accessed via passage through the gastric–gastric fistula to allow ERCP.

In addition to EUS-GJ, a gastrogastric or enterogastric fistula EUS-directed transgastric ERCP (EDGE) can be created to allow access to the excluded stomach in patients with Roux-en-Y gastric bypass. This access can be pursued for various clinical indications but is often established to facilitate ERCP. The first case series of patients undergoing the EDGE procedure was published by Kedia *et al.* [64] in 2015. The process of EDGE is described in Figure 3.

A systematic review of case series and reports documented that the technical success rate of the EDGE procedure has been high (99%), with the excellent success of subsequent ERCP (98%). In this same review, 24% of patients had adverse events. There was 1 case of perforation requiring surgery but 19 other cases of stent migration or maldeployment that could be managed endoscopically. Other adverse events included bleeding (5.3%) and persistent fistula/perforation (<1%) [65].

## Injection therapy

Although EUS has long been used as a diagnostic modality for pancreatic solid and cystic lesions, EUS-directed therapies for these lesions have been slow to gain widespread attention. For patients with potentially malignant mucinous lesions or those with low-grade neuroendocrine tumors, EUS-directed ablation may offer an alternative to surgical alternatives. Studies have demonstrated that direct injection of ethanol can be efficacious. In 2005, Gan *et al.* [66] described a 25-patient case series with EUS-guided ethanol injection into pancreatic cysts. Thirty-five percent of patients had cyst resolution on subsequent imaging. However, this approach seems to predispose patients to pancreatitis (10% of patients with pancreatitis; 13% of patients with abdominal pain). The CHARM trial evaluated the response to treatment in mucinous-type pancreatic cysts that were treated using paclitaxel and gemcitabine with or without alcohol. This prospective trial showed that alcohol was not needed for a cyst response (67% response rate without ethanol vs 61% with ethanol) [67]. Additionally, 6% and 22% of patients experienced major and minor adverse events, respectively, in the ethanol group compared with 0% of adverse events reported in the cyst ablation group without ethanol [67].

## EUS-guided liver biopsy

**Figure 4.**
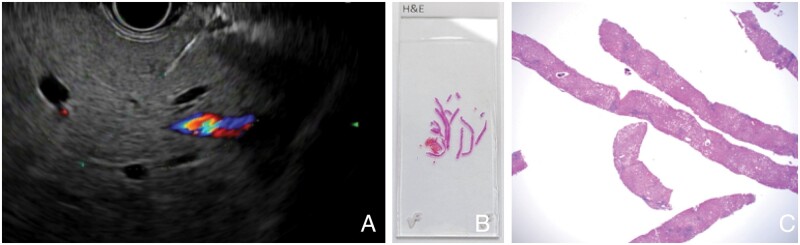
Endoscopic ultrasound (EUS)-guided liver biopsy. (A) A 19-gauge fine-needle biopsy (FNB) needle with transgastric access is used to access the liver. Careful attention is made to avoid any vasculature. Following the biopsy, the needle is slowly withdrawn and the tract is examined for any evidence of bleeding. (B) Gross image from a 19-gauge FNB with excellent core samples with intact portal tracts. (C) ×20 image of FNB core samples.

EUS-guided liver biopsy provides an alternative to percutaneous, transvenous, or laparoscopic liver biopsy (Figure 4). Dewitt *et al.* [68] first described a case series of 21 patients who underwent EUS-guided liver biopsy in 2009. EUS provides several cognitive advantages over traditional liver biopsy. First, EUS allows direct visualization and high-resolution Doppler confirmation of the needle tract. This enables the echoendosonographer to avoid major vasculature and monitor for bleeding after the biopsy (Figure 4A). Additionally, the EUS-guided liver biopsy allows the sampling of both lobes of the liver (Figure 4B and C). Finally, this procedure can be performed concurrently with endoscopy and EUS-guided portal pressure gradient measurement. The major disadvantage of this approach is the potential for inadequate tissue sampling compared with standard percutaneous biopsy. Nevertheless, a recent meta-analysis demonstrated that EUS-guided liver biopsy achieves a successful histologic diagnosis in 93.9% of cases [69]. From a technical perspective, 19-gauge fine-needle biopsy (typically a forked or Franseen-type needle) is superior to traditional aspirates due to better specimen quality (2.09 vs 1.47 cm specimen length) and more complete portal triads (42.6% vs 18.1%) [70]. There have been no observed substantive differences in the rate of adverse events when comparing fine-needle biopsy to aspiration.

## EUS-guided portal pressure gradient measurement

**Figure 5.**
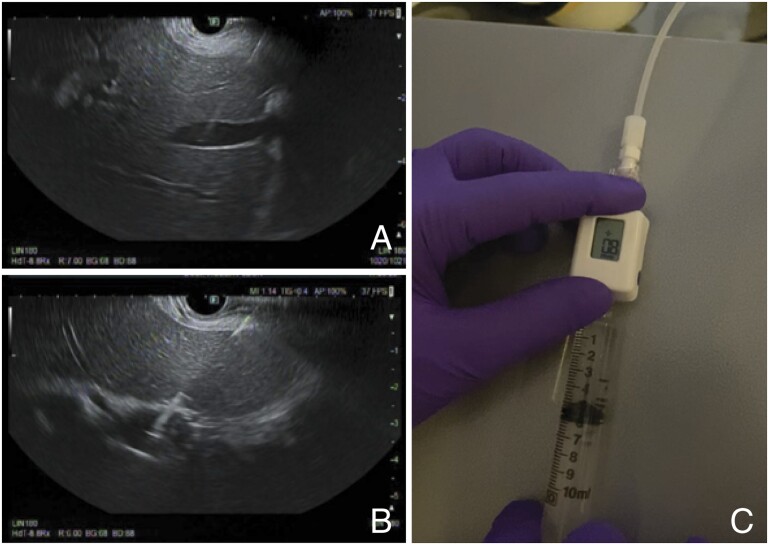
Endoscopic ultrasound (EUS)-guided portal pressure gradient measurement. (A) A 25-gauge needle is used for transhepatic EUS-guided access to the left hepatic vein. (B) The 25-gauge needle is used for transhepatic EUS-guided access to the portal vein. (C) The compact manometer is placed at the midaxillary line to establish the phlebostatic axis.

EUS-guided portal pressure gradient measurement (EUS-PPG) is a novel endosonographic technique allowing the clinician to directly measure the hepatic venous pressure gradient (HVPG). Rather than utilizing wedged hepatic vein pressure as a surrogate for sinusoidal pressure, EUS-PPG directly measures the difference between hepatic and portal vein pressure (Figure 5). Early reports suggest good validity, as EUS-PPG measurements correlate well with HVPG when measured concomitantly in a cohort of patients with known portal hypertension.

There have been several prior studies demonstrating the safety and efficacy of EUS-PPG. Technical feasibility was first shown in porcine models and, in 2014, EUS-PPG was performed successfully in the first human patient [71]. A subsequent case series performed by Huang *et al.* [72] demonstrated an excellent correlation between EUS-PPG-obtained HVPG measurements and endoscopic findings of portal hypertension in 28 patients with suspected cirrhosis. Additionally, two smaller case series showed an excellent correlation between EUS-PPG measurements of HVPG and transjugular (indirect) HVPG, as well as strong associations with liver histology, fibro elastography, and laboratory predictors of fibrosis (fibrosis-4 and non-alcoholic fatty liver disease fibrosis score) [73–76].

Performing EUS-PPG also offers added clinical efficiency, given the concurrent ability to perform esogastroduodenoscopy for the evaluation and treatment of esophageal and gastric varices and/or perform EUS-guided liver biopsy.

## EUS-guided vascular therapy

EUS-guided vascular therapy was first described in 2000 for managing esophageal varices with sclerotherapy. In a case series of five patients, Lahoti *et al.* [77] used a 2.5-mm catheter under EUS guidance to inject sodium morrhuate directly into esophageal varices. There was no reported procedure-related mortality or bleeding. The indications for vascular-related therapies have expanded from this time to treatment of rectal varices and arterial bleeding, management of pseudoaneurysms (endovascular coil placements), embolization of the splenic artery, and portal pressure measurements (discussed previously).

**Figure 6.**
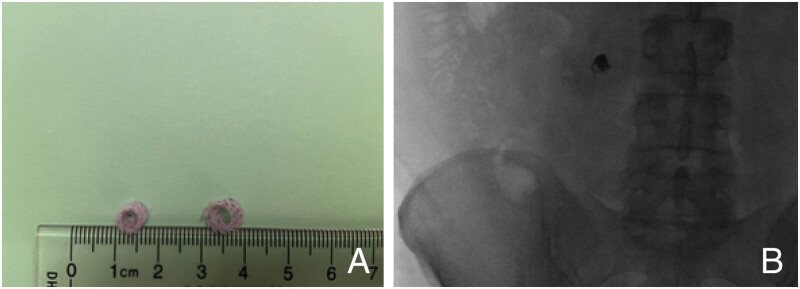
Endoscopic ultrasound (EUS)-guided vascular therapy. (A) The same coils placed transvenously by interventional radiology can also be placed via endoscopic ultrasound. (B) Here we have an endoscopically placed coil in a stomal varices, seen on post-endoscopic abdominal X-ray.

Definitive therapies for gastric varices include injection of cyanoacrylate glue and/or placement of a hemostatic coil within the varix (Figure 6). Placement of the cyanoacrylate glue along with the coil under EUS guidance allows direct visualization of the varix and monitoring for a change in Doppler flow before and after the therapy [78, 79].

EUS management is beneficial in the management of ectopic varices, most commonly with gastric varices. In a single-center retrospective study in 2019 of patients with high-risk gastric varices and recent bleeding, EUS-guided injection of active (or evidence of recent bleeding) gastric varices had less associated rebleeding risk than a direct endoscopic injection of cyanoacrylate glue [80].

In summary, EUS has evolved from a purely diagnostic modality to a sophisticated tool for intervention with numerous luminal, pancreaticobiliary, and hepatic applications. Lumen-apposing stents have revolutionized the field and future development of accessories will propel what can be done endoscopically to a whole new level.

## Authors’ Contributions

All authors contributed to the conception of the manuscript, interpretation of data, critical revision, and final approval of the manuscript.

## References

[1] Strohm WD , Phillip J, Hagenmüller F et al Ultrasonic tomography by means of an ultrasonic fiberendoscope. Endoscopy 1980;12:241–4. 7428729 10.1055/s-2007-1021752

[2] Dimagno EP , Buxton JL, Regan PT et al Ultrasonic endoscope. Lancet 1980;1:629–31. 6102631 10.1016/s0140-6736(80)91122-8

[3] Peery AF , Crockett SD, Murphy CC et al Burden and cost of gastrointestinal, liver, and pancreatic diseases in the United States: update 2021. Gastroenterology 2022;162:621–44. 34678215 10.1053/j.gastro.2021.10.017 PMC10756322

[4] Rogers BH , Cicurel NJ, Seed RW. Transgastric needle aspiration of pancreatic pseudocyst through an endoscope. Gastrointest Endosc 1975;21:133–4. 1112474 10.1016/s0016-5107(75)73821-x

[5] Kozarek RA , Brayko CM, Harlan J et al Endoscopic drainage of pancreatic pseudocysts. Gastrointest Endosc 1985;31:322–7. 4043685 10.1016/s0016-5107(85)72215-8

[6] Sahel J , Bastid C, Pellat B et al Endoscopic cystoduodenostomy of cysts of chronic calcifying pancreatitis: a report of 20 cases. Pancreas 1987;2:447–53. 3628239 10.1097/00006676-198707000-00012

[7] Binmoeller KF , Seifert H, Walter A et al Transpapillary and transmural drainage of pancreatic pseudocysts. Gastrointest Endosc 1995;42:219–24. 7498686 10.1016/s0016-5107(95)70095-1

[8] Kahaleh M , Shami VM, Conaway MR et al Endoscopic ultrasound drainage of pancreatic pseudocyst: a prospective comparison with conventional endoscopic drainage. Endoscopy 2006;38:355–9. 16680634 10.1055/s-2006-925249

[9] Park DH , Lee SS, Moon SH et al Endoscopic ultrasound-guided versus conventional transmural drainage for pancreatic pseudocysts: a prospective randomized trial. Endoscopy 2009;41:842–8. 19798610 10.1055/s-0029-1215133

[10] Wiersema MJ. Endosonography-guided cystoduodenostomy with a therapeutic ultrasound endoscope. Gastrointest Endosc 1996;44:614–7. 8934175 10.1016/s0016-5107(96)70022-6

[11] Wiersema MJ , Baron TH, Chari ST. Endosonography-guided pseudocyst drainage with a new large-channel linear scanning echoendoscope. Gastrointest Endosc 2001;53:811–3. 11375600 10.1067/mge.2001.113272

[12] Borie D , Frileux P, Levy E et al [Surgery of acute necrotizing pancreatitis: active prolonged drainage in 157 consecutive patients]. Presse Med 1994;23:1064–8. 7971817

[13] Baron TH , Thaggard WG, Morgan DE et al Endoscopic therapy for organized pancreatic necrosis. Gastroenterology 1996;111:755–64. 8780582 10.1053/gast.1996.v111.pm8780582

[14] Seifert H , Wehrmann T, Schmitt T et al Retroperitoneal endoscopic debridement for infected peripancreatic necrosis. Lancet 2000;356:653–5. 10968442 10.1016/S0140-6736(00)02611-8

[15] Talreja JP , Shami VM, Ku J et al Transenteric drainage of pancreatic-fluid collections with fully covered self-expanding metallic stents (with video). Gastrointest Endosc 2008;68:1199–203. 19028232 10.1016/j.gie.2008.06.015

[16] Lopes CV , Pesenti C, Bories E et al Endoscopic-ultrasound-guided endoscopic transmural drainage of pancreatic pseudocysts and abscesses. Scand J Gastroenterol 2007;42:524–9. 17454865 10.1080/00365520601065093

[17] Yang D , Perbtani YB, Mramba LK et al Safety and rate of delayed adverse events with lumen-apposing metal stents (LAMS) for pancreatic fluid collections: a multicenter study. Endosc Int Open 2018;6:E1267–75. 30302385 10.1055/a-0732-502 PMC6175687

[18] Vanek P , Falt P, Vitek P et al EUS-guided transluminal drainage using lumen-apposing metal stents with or without coaxial plastic stents for treatment of walled-off necrotizing pancreatitis: a prospective bicentric randomized controlled trial. Gastrointest Endosc 2023;97:1070–80. 36646148 10.1016/j.gie.2022.12.026

[19] Bang JY , Mel Wilcox C, Arnoletti JP et al Importance of disconnected pancreatic duct syndrome in recurrence of pancreatic fluid collections initially drained using lumen-apposing metal stents. Clin Gastroenterol Hepatol 2021;19:1275–81.e2. 32683101 10.1016/j.cgh.2020.07.022

[20] Pawa R , Dorrell R, Russell G et al Long-term transmural drainage of pancreatic fluid collections with double pigtail stents following lumen-apposing metal stent placement improves recurrence-free survival in disconnected pancreatic duct syndrome. Dig Endosc 2022;34:1234–41. 35148447 10.1111/den.14266

[21] Trikudanathan G , Tawfik P, Amateau SK et al Early (<4 weeks) versus standard (≥ 4 weeks) endoscopically centered step-up interventions for necrotizing pancreatitis. Am J Gastroenterol 2018;113:1550–8. 30279466 10.1038/s41395-018-0232-3

[22] Puga M , Consiglieri CF, Busquets J et al Safety of lumen-apposing stent with or without coaxial plastic stent for endoscopic ultrasound-guided drainage of pancreatic fluid collections: a retrospective study. Endoscopy 2018;50:1022–6. 29590668 10.1055/a-0582-9127

[23] Bang JY , Hasan MK, Navaneethan U et al Lumen-apposing metal stents for drainage of pancreatic fluid collections: when and for whom? Dig Endosc 2017;29:83–90. 27199157 10.1111/den.12681

[24] Mukai S , Itoi T, Baron TH et al Endoscopic ultrasound-guided placement of plastic vs. biflanged metal stents for therapy of walled-off necrosis: a retrospective single-center series. Endoscopy 2015;47:47–55. 25264765 10.1055/s-0034-1377966

[25] Bang JY , Navaneethan U, Hasan MK et al Non-superiority of lumen-apposing metal stents over plastic stents for drainage of walled-off necrosis in a randomised trial. Gut 2019;68:1200–9. 29858393 10.1136/gutjnl-2017-315335 PMC6582745

[26] Karstensen JG , Novovic S, Hansen EF et al EUS-guided drainage of large walled-off pancreatic necroses using plastic versus lumen-apposing metal stents: a single-centre randomised controlled trial. Gut 2023;72:1167–73. 36446550 10.1136/gutjnl-2022-328225

[27] Di Mitri R , Amata M, Mocciaro F et al EUS-guided biliary drainage with LAMS for distal malignant biliary obstruction when ERCP fails: single-center retrospective study and maldeployment management. Surg Endosc 2022;36:4553–69. 34724586 10.1007/s00464-021-08808-0

[28] Nennstiel S , Weber A, Frick G et al Drainage-related complications in percutaneous transhepatic biliary drainage: an analysis over 10 years. J Clin Gastroenterol 2015;49:764–70. 25518004 10.1097/MCG.0000000000000275

[29] Gupta K , Perez-Miranda M, Kahaleh M et al; InEBD Study Group. Endoscopic ultrasound-assisted bile duct access and drainage: multicenter, long-term analysis of approach, outcomes, and complications of a technique in evolution. J Clin Gastroenterol 2014;48:80–7. 23632351 10.1097/MCG.0b013e31828c6822

[30] Pizzicannella M , Caillol F, Pesenti C et al EUS-guided biliary drainage for the management of benign biliary strictures in patients with altered anatomy: a single-center experience. Endosc Ultrasound 2020;9:45–52. 31552913 10.4103/eus.eus_55_19 PMC7038727

[31] Sharaiha RZ , Khan MA, Kamal F et al Efficacy and safety of EUS-guided biliary drainage in comparison with percutaneous biliary drainage when ERCP fails: a systematic review and meta-analysis. Gastrointest Endosc 2017;85:904–14. 28063840 10.1016/j.gie.2016.12.023

[32] Kunda R , Pérez-Miranda M, Will U et al EUS-guided choledochoduodenostomy for malignant distal biliary obstruction using a lumen-apposing fully covered metal stent after failed ERCP. Surg Endosc 2016;30:5002–8. 26969661 10.1007/s00464-016-4845-6

[33] El Chafic AH , Shah JN, Hamerski C et al EUS-guided choledochoduodenostomy for distal malignant biliary obstruction using electrocautery-enhanced lumen-apposing metal stents: first US, multicenter experience. Dig Dis Sci 2019;64:3321–7. 31175495 10.1007/s10620-019-05688-2

[34] Attili F , Rimbaş M, Galasso D et al Fluoroless endoscopic ultrasound-guided biliary drainage after failed ERCP with a novel lumen-apposing metal stent mounted on a cautery-tipped delivery system. Endoscopy 2015;47(Suppl 1):E619–20. 26714151 10.1055/s-0034-1393669

[35] Binmoeller KF , Shah J. A novel lumen-apposing stent for transluminal drainage of nonadherent extraintestinal fluid collections. Endoscopy 2011;43:337–42. 21264800 10.1055/s-0030-1256127

[36] Williams EJ , Ogollah R, Thomas P et al What predicts failed cannulation and therapy at ERCP? Results of a large-scale multicenter analysis. Endoscopy 2012;44:674–83. 22696192 10.1055/s-0032-1309345

[37] Irani S , Baron TH, Grimm IS et al EUS-guided gallbladder drainage with a lumen-apposing metal stent (with video). Gastrointest Endosc 2015;82:1110–5. 26142558 10.1016/j.gie.2015.05.045

[38] Teoh AYB , Kitano M, Itoi T et al Endosonography-guided gallbladder drainage versus percutaneous cholecystostomy in very high-risk surgical patients with acute cholecystitis: an international randomised multicentre controlled superiority trial (DRAC 1). Gut 2020;69:1085–91. 32165407 10.1136/gutjnl-2019-319996

[39] Walter D , Teoh AY, Itoi T et al EUS-guided gall bladder drainage with a lumen-apposing metal stent: a prospective long-term evaluation. Gut 2016;65:6–8. 26041748 10.1136/gutjnl-2015-309925

[40] Choi JH , Lee SS, Choi JH et al Long-term outcomes after endoscopic ultrasonography-guided gallbladder drainage for acute cholecystitis. Endoscopy 2014;46:656–61. 24977397 10.1055/s-0034-1365720

[41] François E , Kahaleh M, Giovannini M et al EUS-guided pancreaticogastrostomy. Gastrointest Endosc 2002;56:128–33. 12085052 10.1067/mge.2002.125547

[42] Shami VM , Kahaleh M. Endoscopic ultrasonography (EUS)-guided access and therapy of pancreatico-biliary disorders: EUS-guided cholangio and pancreatic drainage. Gastrointest Endosc Clin N Am 2007;17:581–93. vii-viii. 17640584 10.1016/j.giec.2007.05.015

[43] Falque A , Gasmi M, Barthet M et al Safety and efficacy of EUS-guided pancreatic duct drainage in symptomatic main pancreatic duct obstruction: Is there still a place for surgery? Endosc Int Open 2021;9:e934–42. 34079881 10.1055/a-1302-1484 PMC8159606

[44] Krafft MR , Nasr JY. Anterograde endoscopic ultrasound-guided pancreatic duct drainage: a technical review. Dig Dis Sci 2019;64:1770–81. 30734236 10.1007/s10620-019-05495-9

[45] Basiliya K , Veldhuijzen G, Gerges C et al Endoscopic retrograde pancreatography-guided versus endoscopic ultrasound-guided technique for pancreatic duct cannulation in patients with pancreaticojejunostomy stenosis: a systematic literature review. Endoscopy 2021;53:266–76. 32544958 10.1055/a-1200-0199

[46] Itoi T , Kasuya K, Sofuni A et al Endoscopic ultrasonography-guided pancreatic duct access: techniques and literature review of pancreatography, transmural drainage and rendezvous techniques. Dig Endosc 2013;25:241–52. 23490022 10.1111/den.12048

[47] Siddiqui UD , Levy MJ. EUS-guided transluminal interventions. Gastroenterology 2018;154:1911–24. 29458153 10.1053/j.gastro.2017.12.046

[48] Shimamura Y , Mosko J, Teshima C et al Endoscopic ultrasound-guided pancreatic duct intervention. Clin Endosc 2017;50:112–6. 28391672 10.5946/ce.2017.046 PMC5398367

[49] Thompson CC , Ryou MK, Kumar N et al Single-session EUS-guided transgastric ERCP in the gastric bypass patient. Gastrointest Endosc 2014;80:517. 25028276 10.1016/j.gie.2014.06.011 PMC5038594

[50] Tyberg A , Zerbo S, Barthet M et al A novel technique for salvaging a dislodged lumen-apposing metal stent during creation of an endoscopic gastrojejunostomy. Gastrointest Endosc 2016;83:254. 26264434 10.1016/j.gie.2015.08.003

[51] Itoi T , Tsuchiya T, Tonozuka R et al Novel EUS-guided double-balloon-occluded gastrojejunostomy bypass. Gastrointest Endosc 2016;83:461–2. 26299530 10.1016/j.gie.2015.08.030

[52] Tyberg A , Kumta N, Karia K et al EUS-guided gastrojejunostomy after failed enteral stenting. Gastrointest Endosc 2015;81:1011–2. 25680897 10.1016/j.gie.2014.10.018

[53] Barthet M , Binmoeller KF, Vanbiervliet G et al Natural orifice transluminal endoscopic surgery gastroenterostomy with a biflanged lumen-apposing stent: first clinical experience (with videos). Gastrointest Endosc 2015;81:215–8. 25527056 10.1016/j.gie.2014.09.039

[54] Khashab MA , Kumbhari V, Grimm IS et al EUS-guided gastroenterostomy: the first U.S. clinical experience (with video). Gastrointest Endosc 2015;82:932–8. 26215646 10.1016/j.gie.2015.06.017

[55] Tyberg A , Perez-Miranda M, Sanchez-Ocaña R et al Endoscopic ultrasound-guided gastrojejunostomy with a lumen-apposing metal stent: a multicenter, international experience. Endosc Int Open 2016;4:E276–81. 27004243 10.1055/s-0042-101789 PMC4798937

[56] Perez-Miranda M , Tyberg A, Poletto D et al EUS-guided gastrojejunostomy versus laparoscopic gastrojejunostomy: an international collaborative study. J Clin Gastroenterol 2017;51:896–9. 28697151 10.1097/MCG.0000000000000887

[57] Kouanda A , Binmoeller K, Hamerski C et al Endoscopic ultrasound-guided gastroenterostomy versus open surgical gastrojejunostomy: clinical outcomes and cost effectiveness analysis. Surg Endosc 2021;35:7058–67. 33479837 10.1007/s00464-020-08221-z

[58] Minata MK , Bernardo WM, Rocha RS et al Stents and surgical interventions in the palliation of gastric outlet obstruction: a systematic review. Endosc Int Open 2016;4:e1158–70. 27857965 10.1055/s-0042-115935 PMC5111833

[59] Mehta S , Hindmarsh A, Cheong E et al Prospective randomized trial of laparoscopic gastrojejunostomy versus duodenal stenting for malignant gastric outflow obstruction. Surg Endosc 2006;20:239–42. 16362479 10.1007/s00464-005-0130-9

[60] Jeurnink SM , Steyerberg EW, Van Hooft JE et al; Dutch SUSTENT Study Group. Surgical gastrojejunostomy or endoscopic stent placement for the palliation of malignant gastric outlet obstruction (SUSTENT study): a multicenter randomized trial. Gastrointest Endosc 2010;71:490–9. 20003966 10.1016/j.gie.2009.09.042

[61] Shimura T , Kataoka H, Sasaki M et al Feasibility of self-expandable metallic stent plus chemotherapy for metastatic gastric cancer with pyloric stenosis. J Gastroenterol Hepatol 2009;24:1358–64. 19467141 10.1111/j.1440-1746.2009.05857.x

[62] Khashab MA , Bukhari M, Baron TH et al International multicenter comparative trial of endoscopic ultrasonography-guided gastroenterostomy versus surgical gastrojejunostomy for the treatment of malignant gastric outlet obstruction. Endosc Int Open 2017;5:e275–81. 28382326 10.1055/s-0043-101695 PMC5378550

[63] Bronswijk M , Vanella G, Van Malenstein H et al Laparoscopic versus EUS-guided gastroenterostomy for gastric outlet obstruction: an international multicenter propensity score-matched comparison (with video). Gastrointest Endosc 2021;94:526–36.e2. 33852900 10.1016/j.gie.2021.04.006

[64] Kedia P , Kumta NA, Widmer J et al Endoscopic ultrasound-directed transgastric ERCP (EDGE) for Roux-en-Y anatomy: a novel technique. Endoscopy 2015;47:159–63. 25575353 10.1055/s-0034-1390771

[65] Prakash S , Elmunzer BJ, Forster EM et al Endoscopic ultrasound-directed transgastric ERCP (EDGE): a systematic review describing the outcomes, adverse events, and knowledge gaps. Endoscopy 2022;54:52–61. 33506456 10.1055/a-1376-2394 PMC8783372

[66] Gan SI , Thompson CC, Lauwers GY et al Ethanol lavage of pancreatic cystic lesions: initial pilot study. Gastrointest Endosc 2005;61:746–52. 15855986 10.1016/s0016-5107(05)00320-2

[67] Moyer MT , Sharzehi S, Mathew A et al The safety and efficacy of an alcohol-free pancreatic cyst ablation protocol. Gastroenterology 2017;153:1295–303. 28802565 10.1053/j.gastro.2017.08.009

[68] Dewitt J , Mcgreevy K, Cummings O et al Initial experience with EUS-guided Tru-cut biopsy of benign liver disease. Gastrointest Endosc 2009;69:535–42. 19231495 10.1016/j.gie.2008.09.056

[69] Mohan BP , Shakhatreh M, Garg R et al Efficacy and safety of EUS-guided liver biopsy: a systematic review and meta-analysis. Gastrointest Endosc 2019;89:238–46.e3. 30389469 10.1016/j.gie.2018.10.018

[70] Ching-Companioni RA , Diehl DL, Johal AS et al 19 G aspiration needle versus 19 G core biopsy needle for endoscopic ultrasound-guided liver biopsy: a prospective randomized trial. Endoscopy 2019;51:1059–65. 31342474 10.1055/a-0956-6922

[71] Fujii-Lau LL , Leise MD, Kamath PS et al Endoscopic ultrasound-guided portal-systemic pressure gradient measurement. Endoscopy 2014;46(Suppl 1 UCTN):E654–6. 25526410 10.1055/s-0034-1390845

[72] Huang JY , Samarasena JB, Tsujino T et al EUS-guided portal pressure gradient measurement with a simple novel device: a human pilot study. Gastrointest Endosc 2017;85:996–1001. 27693644 10.1016/j.gie.2016.09.026 PMC5611853

[73] Wadhawan M , Dubey S, Sharma BC et al Hepatic venous pressure gradient in cirrhosis: correlation with the size of varices, bleeding, ascites, and child's status. Dig Dis Sci 2006;51:2264–9. 17080245 10.1007/s10620-006-9310-2

[74] Paik YH. [The relation between hepatic venous pressure gradient and complications of liver cirrhosis]. Korean J Hepatol 2008;14:136–8. 18617760 10.3350/kjhep.2008.14.2.136

[75] Suk KT. Hepatic venous pressure gradient: clinical use in chronic liver disease. Clin Mol Hepatol 2014;20:6–14. 24757653 10.3350/cmh.2014.20.1.6 PMC3992331

[76] Zhang W , Peng C, Zhang S et al EUS-guided portal pressure gradient measurement in patients with acute or subacute portal hypertension. Gastrointest Endosc 2021;93:565–72. 32615178 10.1016/j.gie.2020.06.065

[77] Lahoti S , Catalano MF, Alcocer E et al Obliteration of esophageal varices using EUS-guided sclerotherapy with color Doppler. Gastrointest Endosc 2000;51:331–3. 10699783 10.1016/s0016-5107(00)70363-4

[78] Robles-Medranda C , Oleas R, Valero M et al Endoscopic ultrasonography-guided deployment of embolization coils and cyanoacrylate injection in gastric varices versus coiling alone: a randomized trial. Endoscopy 2020;52:268–75. 32126576 10.1055/a-1123-9054

[79] Henry Z , Patel K, Patton H et al AGA Clinical Practice Update on Management of Bleeding Gastric Varices: Expert Review. Clin Gastroenterol Hepatol 2021;19:1098–107.e1. 33493693 10.1016/j.cgh.2021.01.027

[80] Bick BL , Al-Haddad M, Liangpunsakul S et al EUS-guided fine needle injection is superior to direct endoscopic injection of 2-octyl cyanoacrylate for the treatment of gastric variceal bleeding. Surg Endosc 2019;33:1837–45. 30259158 10.1007/s00464-018-6462-z

